# Impaired Communication Between the Dorsal and Ventral Stream: Indications from Apraxia

**DOI:** 10.3389/fnhum.2016.00008

**Published:** 2016-02-01

**Authors:** Carys Evans, Martin G. Edwards, Lawrence J. Taylor, Magdalena Ietswaart

**Affiliations:** ^1^Faculty of Health and Life Sciences, Department of Psychology, Northumbria UniversityNewcastle upon Tyne, UK; ^2^Institute of Research in the Psychological Sciences, Université catholique de LouvainLouvain-le-Neuve, Belgium; ^3^Psychology, School of Natural Sciences, University of StirlingStirling, UK

**Keywords:** apraxia, visual affordance, ventro-dorsal stream, visual pathways model, grasping

## Abstract

Patients with apraxia perform poorly when demonstrating how an object is used, particularly when pantomiming the action. However, these patients are able to accurately identify, and to pick up and move objects, demonstrating intact ventral and dorsal stream visuomotor processing. Appropriate object manipulation for skilled use is thought to rely on integration of known and visible object properties associated with “ventro-dorsal” stream neural processes. In apraxia, it has been suggested that stored object knowledge from the ventral stream may be less readily available to incorporate into the action plan, leading to an over-reliance on the objects’ visual affordances in object-directed motor behavior. The current study examined grasping performance in left hemisphere stroke patients with (*N* = 3) and without (*N* = 9) apraxia, and in age-matched healthy control participants (*N* = 14), where participants repeatedly grasped novel cylindrical objects of varying weight distribution. Across two conditions, object weight distribution was indicated by either a memory-associated cue (object color) or visual-spatial cue (visible dot over the weighted end). Participants were required to incorporate object-weight associations to effectively grasp and balance each object. Control groups appropriately adjusted their grasp according to each object’s weight distribution across each condition, whereas throughout the task two of the three apraxic patients performed poorly on both the memory-associated and visual-spatial cue conditions. A third apraxic patient seemed to compensate for these difficulties but still performed differently to control groups. Patients with apraxia performed normally on the neutral control condition when grasping the evenly weighted version. The pattern of behavior in apraxic patients suggests impaired integration of visible and known object properties attributed to the ventro-dorsal stream: in learning to grasp the weighted object accurately, apraxic patients applied neither pure knowledge-based information (the memory-associated condition) nor higher-level information given in the visual-spatial cue condition. Disruption to ventro-dorsal stream predicts that apraxic patients will have difficulty learning to manipulate new objects on the basis of information other than low-level visual cues such as shape and size.

## Introduction

Apraxia is a high-level movement disorder that commonly occurs after lesions to the left frontoparietal motor network. In addition to impaired gesture imitation, apraxia is recognized by performance errors when demonstrating how objects are used (Goldenberg, 1995; Buxbaum, 2001). Although these errors are most apparent when pantomiming the use of objects, with a marked improvement during actual object-use, both pantomime and actual use can be affected (De Renzi and Lucchelli, 1988; Buxbaum and Saffran, 2002; Sunderland and Shinner, 2007; Goldenberg, 2009). Skilful manipulation of objects requires the integration of stored information about the object’s typical use and action processes enabling the object to be grasped appropriately based on the object’s visual affordances and spatial location. In the case of apraxia, it is believed that this integrative process is disturbed. However it is currently not clear whether these deficits affect apraxic patients’ ability to learn to manipulate new objects.

Close examination of object knowledge in apraxic patients confirms that performance errors cannot be attributed to impaired ventral (vision-for-perception) or dorsal (vision-for-action) streams of the visual pathways model (Goodale and Milner, 1992; Milner and Goodale, 2006). Apraxic patients can identify visually presented objects (Daprati and Sirigu, 2006) and order familiar objects in weight order (Dawson et al., 2010; Li et al., 2011). These patients also use structural properties to appropriately reach and grasp familiar objects, infer the use of novel objects based on their affordances, and apply appropriate grip force using recent sensorimotor feedback (Gordon et al., 1993; Sirigu et al., 1995; Goldenberg and Hagmann, 1998; Ietswaart et al., 2006; Frey, 2007; Hermsdörfer et al., 2011; Randerath et al., 2011; Sunderland et al., 2013; Eidenmüller et al., 2014). However, patients with apraxia produce incorrect hand postures attributed to functional use of objects and disturbed anticipatory grip force control for familiar objects (Buxbaum et al., 2003). These results confirm that different mechanisms of the visual pathways model are important depending on the goal of the motor act and support recent evidence suggesting that a “ventro-dorsal” sub-stream of the traditional dorsal pathway may be necessary when processing sensorimotor information based on long-term action representations of how objects are functionally used (Buxbaum and Kalénine, 2010; Binkofski and Buxbaum, 2013). It could be that this sub-stream may be implicated in apraxia.

Unlike the dorsal pathway that extends bilaterally from occipital to superior parietal and dorsal pre-motor areas, the ventro-dorsal sub-stream is left lateralized, projecting medially from occipital to left inferior parietal lobe (IPL) and ventral pre-motor regions. Through a mutual connection with the ventral stream via the left IPL, perceptual information can be incorporated into action plans (Rizzolatti and Matelli, 2003; Buxbaum and Kalénine, 2010; Rizzolatti et al., 2011; Binkofski and Buxbaum, 2013; Vingerhoets, 2014) enabling objects to be grasped for use by applying stored knowledge of how objects are functionally manipulated to the physical properties of the objects presented (Frey, 2007; Almeida et al., 2013; Garcea and Mahon, 2014). In support of object-use errors observed in apraxia, there is an established relationship between apraxic symptoms and damage to regions implicated in the ventro-dorsal stream, in particular inferior parietal regions that suggest this pathway may indeed be disrupted (Haaland et al., 2000; Buxbaum, 2001; Buxbaum et al., 2006, 2007; Frey, 2007; Goldenberg, 2009; Garcea and Mahon, 2014). The subsequent failure to effectively access and implement information from the ventral stream into the action plan results in an over-reliance on the intact dorsal stream. Consequently, objects are manipulated based on what is visually afforded irrespective of the goal of the action (Randerath et al., 2011).

That said, apraxic patients have shown equivalent performance to controls when making memory-driven reach and grasp movements also reliant on the integration of ventral and dorsal processes (Ietswaart et al., 2001; Dawson et al., 2010). Although these findings suggest that apraxic patients can successfully utilize stored representations, it remains possible that the visuo-motor transformation involved in simple reach and grasp movements may not be difficult enough to place sufficient demand on high-level perceptual processes. The proposal of ventro-dorsal disturbance in apraxia has also been argued to place too much importance on different components of object knowledge; in particular, retrieval of knowledge of an objects prototypical use that is dependent on previous experience, which cannot account for apraxic errors during novel object-use (Goldenberg and Hagmann, 1998; Goldenberg, 2014). Yet such knowledge retrieval furthermore assumes that skilled object-use relies on the retrieval of information from “storehouses” as opposed to the convergence of short- and long-term visual representations depending on the goal of the motor act.

While the research outlined suggests apraxic patients have difficulties accessing and incorporating stored knowledge of actions related to skilled use of familiar objects, it remains unclear how these patients learn to manipulate new objects. Of the few studies that have assessed this issue, Barde et al. (2007) trained patients to match novel gestures to novel object pictures that were high or low afforded by associated objects. Apraxic patients demonstrated a greater ability to correctly match gestures to object shape for the high than low afforded gestures during action recognition, but were consistently poor compared to controls during action production regardless of affordance. This may be due to the use of two-dimensional objects during training reducing the affordance bias during action production. Retrieval of the appropriate action associated with the object may also have been more difficult when the goal was simply to produce the correct action, as there is no clear feedback as to whether the action goal was achieved in a comparable manner to appropriately grasping an object to fulfil a function.

The current study explored the impact of affordance on object manipulation by requiring participants to repeatedly lift and balance novel objects of differing weight distribution. Over two conditions, the weight distribution of different cylindrical objects was indicated using different object-weight associations, either by a symbolic memory-association between the color of the object and its weight distribution or by a visual-spatial cue of a “dot” over the weighted end of the object. Change in object manipulation over repeated lifts determined whether apraxic patients successfully used object knowledge obtained through experience to inform their grasp, or whether they continually relied on the visual cues to guide action.

Specifically, this study examined participants’ point of grasp along the object depending on weight distribution. When grasping unbalanced objects, healthy adults intuitively choose a grasp close to the center of mass in order to minimize the energy required by grip force to compensate for load torque (Salimi et al., 2003; Duemmler et al., 2008; Endo et al., 2011). This is said to be estimated visually prior to initial object grasping, which is reflected in accurate grasping of unfamiliar objects for the first time (Lederman and Wing, 2003) or when asked to visually point to the center of mass (Baud-Bovy and Soechting, 2001; Duemmler et al., 2008). Action execution was used throughout the study rather than perceptual task learning. This enabled apraxic patients to get strong visual feedback as to whether the action goal of balancing each object had been achieved during each trial. It was anticipated that apraxic patients would show greater performance accuracy when the object afforded the correct gesture with increased contextual information provided (akin to findings by Barde et al. (2007) in the recognition task).

During the memory-associated condition, when each object’s weight distribution was indicated symbolically by the color of the object, apraxic patients were expected to be impaired. Due to the symmetrical shape of the object, apraxic patients were expected to be biased towards more central grasp points and require a greater number of trials to accurately balance the object. In the visual-spatial cue condition, when the center of mass is indicated by a “dot” over the weighted end, apraxic patients may benefit from this meaningful visible cue over time to prompt a more accurate grasp-point over each trial. An alternative prediction was that apraxic patients might continue to use low-level affordance cues of object structure to indicate weight distribution, resulting in more central grasps rather than to the left or right of the object. Inappropriate manipulation of memory-associated and visual-spatial cued objects would confirm that apraxics over-rely on visual information processed by the dorsal visual stream due to ventral, stored knowledge, being unsuccessfully incorporated into the action plan via the ventro-dorsal sub-stream. Such behavior would add insight into what information apraxic patients can effectively utilize during goal directed action.

## Methods

### Participants

Twenty-seven right-handed participants were recruited, 13 of which had suffered a stroke (*M*_age_ 68 ± 14, 8 male) within 27 months (*M*_months_ 15 ± 10) and 14 age-matched healthy control participants (*M*_age_ 70 ± 9, 5 male). In the patient group, and at the time of testing, three patients displayed symptoms of apraxia and 10 patients did not show signs of apraxia. The ethics committee within Northumbria University’s Department of Psychology and a local NHS ethics committee approved the project.

**Figure 1:**
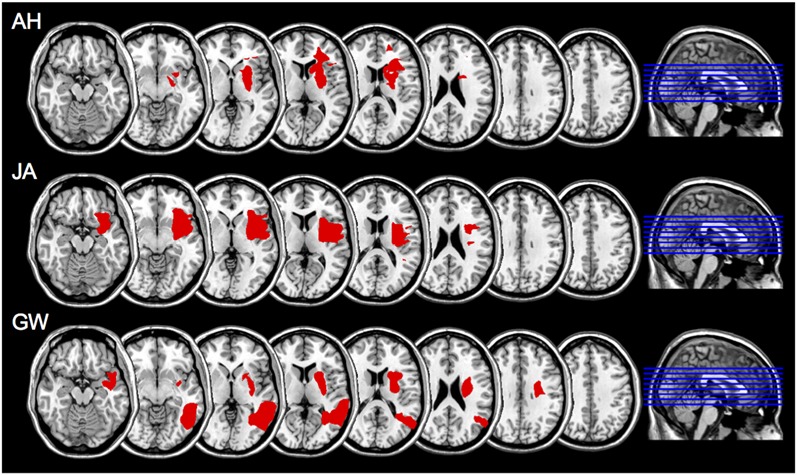
**Scan slices for apraxic patients AH, JA, and GW; lesioned areas were applied to a template scan allowing clear visualization of the anatomical landmarks.** The lesion area(s) are in red. Left is right as per neurological convention.

On the basis of CT, MRI scans and clinical notes, patients who had a brain hemeorrhage or an infarct involving the left hemisphere were recruited from rehabilitation centers and National Health Hospitals within the North East of England. Patients presented with degrees of aphasia, right-sided weakness, or sensory loss. Table 1 describes each patient’s lesion and the Brodmann areas implicated. Lesions were mapped using MRIcron software package (Rorden et al., 2007)^1^ based on the radiologist’s MRI and/or CT clinical scans of each patient. The areas of damage for each patient were mapped using MRIcron software package; lesions were determined based on the radiologist’s scan reports and the digital brain image. Scans were then normalized to a common stereotaxic space using Clinical Tool box software through SPM and applied to the Brodmann Atlas included in MRIcron (Rorden et al., 2012)^2^. Lesions for the three apraxic patients are visually documented in Figure 1.

**Table 1:** **Description of each apraxic (top) and non-apraxic (bottom) patient’s lesion as described in the radiologist’s CT and/or MRI reports and when mapped onto the Brodmann atlas**.

			Brodmann areas damaged (% = amount lesioned)		
Patient	Includes IPL	Lesion—left hemisphere lesion information on basis of acute CT/MRI report	>75%	25–75%	<25%
AH	N	L MCA infarct involving L putamen, internal capsule, and caudate head. Extending into L frontal white matter.	34		10, 11, 25, 32, 47, 45, 46
GW	Y	L temporo-parietal, basal ganglia, and parieto-occipital infarcts.		22, 31, 37, **39**	6, 19, 20, 34, 36, 38
JA	N	L MCA infarct.	34, 38	47	6, 11, 20, 21, 22, 41, 44
SG	N	L corona radiata infarct.			
TY	N	L frontal MCA infarct.		47	11, 38
DF	-	L fronto-temporo-parietal infarct and L insula.			
WM	-	L total anterior circulation infarct.			
MB	N	L frontal lobe, thalamus, lentiform, R caudate head, bilateral basal ganglia lacunar infarcts.			
TM	N	Ischemeic change in the L MCA occlusion.			42
DJ	N	L frontal MCA infarct.	44	6, 38, 43	9
JS	N	Mild white matter ischemeic change.			
BH	N	L thalamus bleed.			

*Note: F, Female; M, Male; Y, Yes; N, No; L, Left; R, Right; ACA, Anterior Cerebral Artery; MCA, Middle Cerebral Artery. Brodmann areas ascribed to the IPL, inferior parietal lobe (areas 39 and 40) are indicated in bold*.

The presence of apraxia was classified on the basis of abnormal performance in one or more of the apraxia screening tools assessing gesture imitation and familiar object-use (pantomime and actual use). Further test batteries and clinical notes were used to exclude any patient presenting with global cognitive deficits or known dementia, severe receptive aphasia or failure to follow one-stage commands (according to the language comprehension token test by De Renzi and Faglioni, 1978), or significant signs of visuospatial neglect (according to the Apples Test by Bickerton et al., 2011). One non-apraxic patient was later excluded (FR) as he was diagnosed with early onset of vascular dementia. Patient details are described in Table 2 and apraxia screening performance in Table 3.

**Table 2:** **Screening performance of patient groups, including apraxics (top) and non-apraxics (bottom); includes FR who was excluded due to early onset vascular dementia**.

Patient	Sex	Age at test (years)	Days post stroke at test	Right sided motor weakness admission	Aphasia noted on admission	Neglect/hemianopia	Language comprehension (stage reached of Token Test)
AH	F	72	226	Y	Y	R neglect	6
GW	M	49	87	Y	Y	n.t.	3
JA	F	48	486	Y	Y	N	2
SG	F	66	833	Y	Y	N	6
TY	M	76	783	N	Y	N	5
DF	M	70	754	Y	Y	N	6
WM	M	78	152	Y	N	N	6
MB	F	49	142	Y	Y	N	6
TM	M	61	169	Y	Y	N	6
DJ	M	84	130	N	Y	N	5
JS	F	91	823	Y	N	N	6
BH	M	58	843	Y	N	N	6

*Note: F, Female; M, Male; Y, Yes; N, No; L, Left; R, Right; n.t, not tested*.

**Table 3:** **Apraxia screening performance and error types in apraxics (top) and non-apraxics (bottom)**.

	Apraxia screening							
	Gesture imitation (total score)				Object use (total score)			
Patient	Hand (20)	*Errors*	Fingers (20)	*Errors*	Pantomime (53)	*Errors*	Actual (18)	*Errors*
AH	19	*fe*	19	*fe*	37	*bpo; ss; gm*	18	
GW	16	*hm; sm*	4	*p of hands; sm*	10	*ao; aa*	16	*aa*
JA	19	*sm*	20		36	*bpo; ss; gm; sm*	16	*ss; sm*
SG	20		20		53		18	
TY	18	*sm*	18	*sm*	48	*bpo; sm*	18	
DF	18	*hm*	20		50	*gm; sm*	18	
WM	20		20		48	*gm; sm*	18	
MB	19	*hm*	19	*sm*	53		18	
TM	20		20		53		18	
DJ	18	*hm*	19	*fe*	53		18	
JS	20		20		53		18	
BH	20		20		51	*ss*	18	

*Note: Types of performance error were given the following acronyms: gesture imitation: perseveration (p); hand misorientation (hm): misorientation of the hand relative to the face; finger extension (fe): incorrect fingers extended from hand; spatial misorientation (sm): hand misorientation relative to the experimenter, e.g., back of hand instead of palm facing. Object use: action addition (aa): miscellaneous actions not interpretable as a step in the task, e.g., waving; action omission (ao): failed to perform any recognisable action; step omission (so): failed to complete some parts of the movement, e.g., rotating hand when squeezing a lemon; body-part-as-object (bpo): e.g., brush teeth with finger; semantic substitution (ss): e.g., stir with fork; grasp misestimation (gm): incorrect grasp size/type for object, e.g., pincer grip for cup; spatial misestimation (sm): incorrect relationship between object relative to body or another (reference) object*.

Healthy age-matched control participants did not have a history of brain damage or stroke. These participants were recruited from the Psychology Department’s participant database and were given monetary compensation for their time.

### Materials

#### Apraxia Screening

##### Gesture imitation of hand and finger postures (Goldenberg, 1996)

The experimenter demonstrated different hand postures relative to the head and finger postures irrespective of the hands position in relation to the body. Gestures were performed “like a mirror”; the experimenter sat opposite the patient, performing each posture with their right hand to be imitated by the patients’ left hand after the demonstration had ended. Successful imitation of each gesture on the first trial was awarded two points; one point was given if the patient was successful after a further demonstration; zero points if the gesture was not imitated correctly. A total score of 20 could be achieved by imitating 10 gestures of each kind.

##### Pantomime of object use (based on Goldenberg et al., 2007)

Participants were required to demonstrate the use of 19 objects. The experimenter presented a drawn image of each object (taken from Cycowicz et al., 1997) and named the action to be pantomimed. Points were given for the presence of predefined movement features (Goldenberg et al., 2007 details these). With exception to demonstrating the use of scissors, body-part-as-object errors were marked as incorrect. A total of 53 points could be obtained, with less than 43 measured as pathological.

##### Actual object use (based on De Renzi and Lucchelli, 1988)

Participants were given the same verbal description of the action to be demonstrated as in the pantomime task. Eighteen of the pantomimed objects were presented; one point was given if used correctly and zero if incorrect. The incorrect use of two or more objects was considered pathological.

#### Object Grasping Task

##### Object stimuli

Five cardboard cylinder tubes (length: 24.5 cm, diameter: 3.7 cm) were used, each containing a 17 g weight (length: 2 cm, diameter: 1.5 cm) in one or both ends. The five cylindrical objects comprised of two experimental conditions: “memory-associated” and “visual-spatial cue”, and one screening condition: “neutral-control”. The “memory-associated” condition consisted of one green and one blue cylinder; when presented to the participant, the green object was weighted on the left, whereas the blue object was weighted on the right. Participants were required to remember the color-weight associations when lifting the object without a visual cue indicating weight distribution on either end of the cylinder. The visual-spatial cue condition consisted of two gray objects that were unevenly weighted, containing a weight in either the left or right end of the object. The heavier end of each object was marked with a red “dot” (1 cm diameter), which acted as a visual cue of the weight distribution when acting upon the object. Finally, the neutral-control condition consisted of one gray object that was evenly weighted with one weight in each end of the cylinder. This screened for any confounds such as visuospatial neglect or comprehension issues that would impact task performance. In addition to the main objects, two white practice cylinders were used when giving task instructions: one evenly-weighted (length: 42 cm, diameter: 1.5 cm) and one unevenly-weighted object (length: 46, diameter 1.7 cm, 34 g weight on the right side). The practice cylinders did not resemble test objects in size and weight to minimize priming effects of grasping these objects prior to the main experiment.

**Figure 2:**
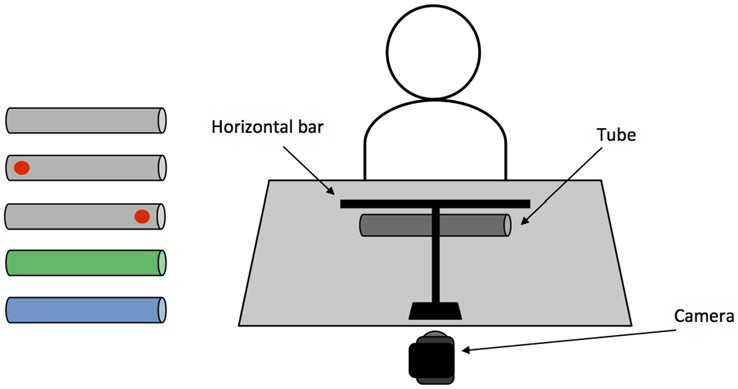
**(Left)** Objects used in the main task. From top: neutral-control evenly weighted; left and right weighted visual-spatial “dot” cue; left weighted/green and right weighted/blue memory-associated. **(Right)** Schematic representation of the experimental setup.

A horizontal bar (length: 30 cm, diameter: 0.5 cm) was positioned perpendicular to the participant, 35 cm in front of the participant and 24 cm above the table. Both the experimenter and participant used the bar to indicate the extent to which the object was balanced. For the duration of testing a video camera was placed behind the horizontal bar and recorded each trial. A schematic representation of the experimental setup can be seen in Figure 2.

### Procedure

Each participant was seated at the workspace where the objects were presented. Using the horizontal bar as a guide, participants were instructed to lift and balance each object using a pincer grip with the index and thumb of their left hand. After the object was lifted to the horizontal bar, participants returned the object to the table and removed their hand from it before another trial began. It was emphasized that if the object was imbalanced, they should not compensate by tightly pinching the object or rotating their wrist during or at the end of each lift. Task instructions were demonstrated using the evenly weighted practice cylinder. Participants were then requested to practice the task procedure using the same cylinder. Once participants successfully completed the movement they were presented the unevenly weighted practice cylinder and repeated the process. After it was evident that participants understood the procedure, the main task was started. During the main task, to ensure each participant had the same experience with the object, they were asked to lift and balance each object five times before being presented the next object. In each block, objects were presented in a random order. Overall, there were five testing blocks in which participants saw each object once; including each individual trial, participants lifted each object 25 times, totalling 125 trials. The video camera recorded participants completing each trial.

### Data Analysis

Task performance across each condition was initially compared between each control group (healthy and non-apraxics) using a two-way mixed model ANOVA exploring OBJECT (memory-associated; visual-spatial cue; neutral-control) × GROUP (Healthy vs. Non-apraxic controls) to rule out differences across control groups. Each apraxic patient was then compared to the control groups separately using modified *t*-tests recommended when estimating the abnormality of an individual patient’s score against a control sample that is modest in size (Crawford and Garthwaite, 2002; Crawford et al., 2010). In order to purely assess whether object-weight associations were learnt as opposed to participants relying on semantic labelling (e.g., green is left weighted) to complete the task, object-weight associations were not explicitly described to the participants during the study. This also accommodated for any language deficits. Instead, learning of object-weight associations was determined by assessing participants’ change in performance accuracy over trials (TC) and change in performance accuracy over blocks (BC). The former would indicate whether apraxic patients’ performance improved with repeated lifts of the same object and the latter would confirm whether apraxic patients applied what they had learned in previous blocks when each object was reintroduced. The points at which the object was grasped were used as a guide to evaluate grasp behavior.

Firstly, in order to analyze the video footage, photo snapshots were created when participants were at the maximal point of object lift. From each snapshot, the “point of grasp” was measured based on the midpoint position of the index finger along the object (from right to left). Grasps were considered accurate depending on whether the object was successfully balanced and an appropriate point of grasp was applied to compensate for the objects weight distribution. This ensured that participants were accurate due to adjusting their grasp-point along the object, as opposed to applying greater grip force or by rotating their wrist during each lift. If the location of an individual’s grasp was greater than two standard deviations from the “optimum” point of grasp (OP) to compensate for weight distribution, it was marked as inaccurate. The optimum point of grasp was measured for each object based on healthy control participants mean point of grasp for the fifth trial across all blocks.

#### Accuracy Change Over Trials (TC)

Grasp accuracy was compared between Trial 1 and Trial 5 across blocks. Performance change across trials would indicate whether apraxic patients’ performance improved with repeated grasps of the same object. To compare performance, accuracy was first weighted; accurate grasps in early trials (e.g., Trial 1) received a greater weighting compared to accurate grasps in later trials (e.g., Trial 5). This reflected the extent to which performance was driven by trial-and-error or learning each objects weight distribution. Inaccurate grasps were given a negative score: fewer points were deducted when grasps were inaccurate in early trials and greater points deducted when performing inaccurately in later trials. These reflected the extent to which participants failed to adapt their grasp based on each objects’ weight distribution with repeated grasps of the same object (see Table 4 for weighted scores). As a greater score could be achieved in Trial 1 compared to Trial 5, these scores were then calculated as proportions of the maximum score achievable in that trial, across all 5 blocks. For example, in Trial 1 an accurate grasp scores 5 points, over 5 blocks a maximum score of 25 can be achieved, whereas for Trial 5 an accurate grasp scores 1 point, over 5 blocks a maximum score of 5 can be achieved. Once participants’ scores in Trial 1 and Trial 5 were transformed into proportions, accuracy in Trial 5 was deducted from Trial 1 (as outlined in the equation below). Based on this calculation, a greater negative score signifies improved accuracy across trials, a positive score signifies reduced or consistently poor performance across trials, and a score of zero indicates that the participant achieved the highest accuracy across trials.

**Table 4:** **Weighted scores for analyses of accuracy change over Trial and Block**.

	1	2	3	4	5
**Trial**
Correct	5	4	3	2	1
Incorrect	−1	−2	−3	−4	−5
**Block**
Correct	5	4	3	2	1
Incorrect	−1	−2	−3	−4	−5

Accuracy change (TC) = (block 1–5 average score^trial 1^/maximum score^trial 1^) − (block 1–5 average score^trial 5^/maximum score^trial 5^).

#### Accuracy Change Over Blocks (BC)

Using the same calculation, performance across blocks was assessed by comparing the average accuracy across trials between Block 1 and Block 5. Performance change across blocks would confirm whether apraxic patients applied what they had learned in previous blocks when each object was reintroduced. As with trial data, performance across blocks was weighted using positive and negative scores. In early blocks, participants received greater points for accurate grasps and fewer points were deducted for inaccurate grasps, whereas in later blocks participants received fewer points for accurate grasps and more points were deducted for inaccurate grasps. Scores were transformed into proportions of the maximum score before accuracy in Block 5 was deducted from accuracy in Block 1.

Notably during testing, non-apraxic patients BH and JS completed only four testing blocks due to experiencing fatigue when lifting the objects several times. The same calculation applied to the final block was instead applied to Block 4 for these patients.

## Results

In order to confirm whether apraxic patients utilized memory-associations or visual-spatial cues regarding weight distribution when balancing each object, performance change across trials and across blocks were assessed. Points of grasp for each object were used as a guide to evaluate grasp behavior.

### Accuracy Change Across Trials (TC)

#### Healthy Controls vs. Non-Apraxics

An initial two-way mixed model ANOVA exploring OBJECT (memory-associated; visual-spatial cue; neutral-control) × GROUP ruled out differences in performance change across Trials in healthy and non-apraxic controls. Non-significant main effects confirmed that performance was comparable across control groups (GROUP: *F*_(1,21)_ = 0.139, *p* = 0.713, ${\text{η}}_{p}^{2}$ = 0.007) and between objects (OBJECT: *F*_(1.357,28.504)_ = 3.583, *p* = 0.058, ${\text{η}}_{p}^{2}$ = 0.145). However, a significant interaction OBJECT × GROUP (*F*_(1.357,28.504)_ = 8.479, *p* = 0.004, ${\text{η}}_{p}^{2}$ = 0.288) was identified. Independent samples *t*-test did not reveal significant differences in performance for all conditions (*p* > 0.05) except the neutral-control condition (*t*_(21)_ = 2.353, *p* = 0.028). Non-apraxics showed greater improvement in task performance from Trial 1–5 (TC = −0.333 ± 0.280) on the evenly weighted object compared to healthy controls whose performance reduced (TC = 0.257 ± 0.714). Notably, differences easily arise on the evenly-weighted neutral-control object, because the point scoring system works with difference from the mean and standard deviation on this condition in normal performance is very small (and differences are therefore of limited interest).

**Figure 3:**
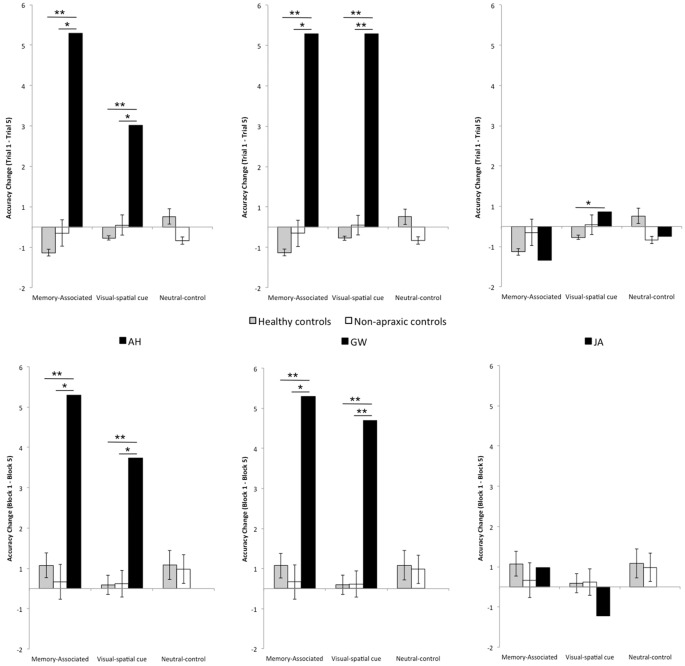
**(Top)** Change in grasp accuracy between Trial 1 and Trial 5 across blocks, including standard error bars. **(Bottom)** Change in grasp accuracy between Block 1 and Block 5 across trials, including standard error bars. For both Trial and Block analyses a negative score indicates an improvement in performance across trials; a positive score indicates a reduced or consistently poor performance. Scores close to zero reflect consistent high accuracy across trials. The black bars at the top of the graphs indicate significant relationships: two asterisks denotes a *p* value < 0.001, and a single asterisk denotes a *p* value < 0.05.

Despite variances in performance change for the neutral-control object, healthy and non-apraxic controls consistently grasped the object close to the optimum grasp-point (OP = 13.18 cm). Examining grasp-point behavior of controls across all three conditions, both groups initially grasped closer to the center of each object in Trial 1, but by Trial 5 were ≤1.32 cm from the optimum grasp-point for each object. Observing individual scores for performance change over trials (TC) confirms that each control participant appropriately adapted their grasp-point over repeated lifts to account for the weight distribution of each object. Of note, non-apraxic control participant JS did not perform as efficiently as the other non-apraxic patients in the memory-associated and visual-spatial cue conditions. However, she was still markedly more accurate than AH and GW. Patient JS also performed at ceiling during the language comprehension test and apraxia screening indicating that her performance was not applicable to poor comprehension or apraxia. Instead, her performance may be more attributable to her age; JS was the oldest participant (91) and testing had to be terminated after the fourth test block as she became fatigued. Together, these findings indicate that healthy and non-apraxic controls effectively utilize both memory-associated and visual-spatial cued information to improve performance when repeatedly lifting each object (see Table 5 for performance change over trials, Table 6 for participants’ average points of grasp, and Figure 3 for accuracy change across trials).

**Table 5:** **Performance change over trials (TC) and blocks (BC) in non-apraxic (top) and apraxic (bottom) patients**.

	Change across trials (TC)			Change across blocks (BC)		
PT	Memory-associated	Visual-spatial cue	Neutral-control	Memory-associated	Visual = spatial cue	Neutral-control
SG	−0.48	−0.24	−0.24	−0.36	0.48	0
TY	1.2	0.6	0	0	0.24	0
DF	−0.48	−0.12	0	−0.24	−0.12	0
WM	−0.84	−0.165	−0.48	2.16	0.28	1.2
MB	−0.6	−0.84	−0.48	−0.24	0.12	1.92
TM	−0.96	−0.24	−0.48	0.36	−0.12	0
DJ	−0.12	0.36	−0.72	0	−0.36	1.2
JS	1.8	1.65	0	1.8	1.65	−1.5
BH	−0.9	−0.6	−0.6	−1.99	−1.11	1.5
	
*M*	−0.153	0.045	−0.333	0.166	0.118	0.48
	
AH	4.8	2.52	0	4.8	3.24	0
GW	4.8	4.8	0	4.8	4.2	0
JA	−0.84	0.36	−0.24	0.48	−0.72	0

*Note: M, mean*.

**Table 6:** **Point of grasp (cm).** Top: Trial 1 and 5 across blocks, including the overall average point of grasp and standard deviation across every trial for each object. Bottom: Block 1 and 5 across trials, including the overall average point of grasp and standard deviation across every block for each object.

	Point of Grasp (distance from OP)									
	Memory-associated				Visual-spatial cue (Dot)				Neutral-control	
	Left weighted (OP = 20.18)		Right weighted (OP = 6.30)		Left weighted (OP = 19.85)		Right weighted (OP = 6.29)		Evenly weighted (OP = 13.18)	
	1	5	1	5	1	5	1	5	1	5
**Trial**
AH	11.50 (8.69)	12.55 (7.63)	12.00 (−6.83)	11.35 (−6.18)	11.75 (8.10)	12.00 (7.85)	12.00 (−5.70)	11.10 (−4.80)	11.70 (1.48)	11.55 (1.63)
GW	13.70 (6.49)	15.00 (5.18)	13.60 (−8.43)	13.55 (−8.38)	13.65 (6.20)	13.95 (5.90)	12.95 (−6.65)	13.00 (−6.70)	13.30 (−0.12)	13.60 (−0.42)
JA	17.10 (3.09)	21.30 (−1.12)	15.70 (−10.53)	2.55 (2.62)	20.70 (−0.85)	18.54 (1.31)	5.55 (0.75)	2.10 (4.20)	14.30 (−1.12)	12.85 (0.33)
Healthy
Controls	14.09 (6.10)	20.21 (−0.03)	11.53 (−6.36)	5.15 (0.02)	17.48 (2.37)	19.84 (0.01)	9.60 (−3.31)	6.30 (0)	13.48 (−0.29)	13.18 (0.01)
Non-apraxics	13.48 (6.80)	19.04 (1.22)	11.26 (−6.07)	5.62 (−0.52)	16.45 (3.45)	19.05 (0.89)	9.23 (−3.01)	5.88 (0.33)	11.91 (1.33)	12.57 (0.58)
**Block**
AH	12.10 (8.08)	13.45 (7.30)	11.70 (−6.53)	12.60 (−7.43)	11.80 (8.05)	12.55 (7.30)	11.75 (−5.45)	11.50 (−5.20)	11.70 (1.48)	11.70 (1.48)
GW	15.65 (4.53)	15.40 (4.45)	13.95 (−8.78)	14.35 (−9.18)	14.10 (5.75)	15.40 (4.45)	13.50 (−7.20)	13.90 (−7.60)	12.70 (0.48)	14.95 (−1.77)
JA	20.85 (−0.67)	20.80 (−2.10)	6.55 (−1.38)	4.80 (0.37)	6.74 (13.11)	21.95 (−2.10)	5.70 (0.60)	2.20 (4.10)	12.60 (0.58)	12.65 (0.53)
Healthy controls	17.98 (2.20)	19.32 (−0.04)	7.43 (−2.25)	6.28 (−1.11)	16.66 (3.19)	19.89 (−0.04)	7.80 (−1.51)	6.58 (−0.28)	12.86 (0.32)	12.99 (0.19)
Non-apraxics	16.93 (3.25)	18.96 (0.50)	8.86 (−3.39)	5.21 (−0.58)	16.47 (3.39)	19.77 (0.50)	7.69 (−1.39)	5.37 (−0.01)	13.10 (0.08)	11.37 (1.32)

*Note: OP, optimum grasp-point to compensate for objects’ weight distribution*.

#### Patient AH

Single case *t*-tests confirmed that when grasping memory-associated objects, patient AH was significantly worse than healthy (*p* < 0.001, *t* = 17.100) and non-apraxic controls (*p* = 0.001, *t* = 4.775) with at least a minimum of 99.93% of controls falling below AH’s score. During the visual-spatial cue condition, patient AH also performed significantly worse than both healthy controls (*p* < 0.001, *t* = 13.363) and non-apraxics (*p* = 0.007, *t* = 3.160) with at least a minimum of 99.33% of controls falling below AH’s score. For both memory-associated and visual-spatial cue conditions, AH’s accuracy was consistently poor (TC ≥ 2.52) whereas control groups generally improved performance across trials (TC from 0.045 to −0.274).

Observing the average grasp-points for both the memory-associated and visual spatial cue conditions, patient AH maintained a point of grasp towards the center of each object (from 11.10 cm to 13.45 cm). These grasps were at least 4.8 cm from the optimum grasp-point to compensate for weight distribution of each object. Unlike control groups, patient AH did not adjust her grasp towards the weighted end of across trials.

As this patient did not adjust her grasp away from the midpoint, when grasping the neutral-control object AH’s performance change was comparable to both healthy controls (*p* = 0.367, *t* = −0.348; an estimated 36.68% falling below AH’s score) and non-apraxics (*p* = 0.271 *t* = 1.128; an estimated 85.40% falling below AH’s score). AH’s use of midpoint grasps confirms that her symptoms of right-sided visual neglect identified in the cancellation task did not affect grasp performance.

#### Patient GW

Performance of patient GW mirrored that of patient AH. Performance change over trials was worse than healthy and non-apraxic controls when grasping unevenly weighted objects in both the memory-associated and visual-spatial cue conditions: for all comparisons *p* ≤ 0.001, with at least an estimated 99.93% of controls falling below GW’s score. Patient GW was consistently unsuccessful in balancing these objects (TC = 4.8 for each), with average points of grasp ranging from 13.46 cm to 14.76 cm across all four objects, and at least 5.18 cm from the optimum grasp-point. Overall, GW’s average grasp was consistently close to or slightly to the left of each object’s center regardless of their weight distribution, with minimal variance in grasp-points across conditions. However when grasping the neutral-control object, GW’s performance was comparable to both healthy (*p* = 0.367; an estimated 36.68% falling below GW’s score) and non-apraxic controls (*p* = 0.146; an estimated 85.40% falling below GW’s score). Patient GW’s average grasp-points were close to the optimum point of grasp.

#### Patient JA

Apraxic patient JA’s performance change across trials was comparable to both healthy and non-apraxic controls for the memory-associated and neutral-control conditions (*p* > 0.05; an estimated 25.65% to 61.96% of controls falling below JA’s score). During the visual-spatial cue condition, although JA was comparable to non-apraxics (*p* = 0.349, *t* = 0.402; an estimated 65.10% of controls falling below JA’s score), performance change was significantly different to healthy controls (*p* = 0.005, *t* = 3.032; an estimated 99.52% of controls falling below JA’s score). It was evident in this condition that JA did not greatly improve grasp accuracy between Trial 1–5 (TC = 0.360) and continued to make errors by the final trial. Although JA achieved largely normal performance on this measure of accuracy change across trials, her qualitative behavior did not look normal. She was slow and deliberate in her reach movements, apparently in an attempt to compensate for her difficulty performing this task. This prompted a closer look at grasp-point and grasp-point variance, in an attempt to quantify her unusual behavior in performing the task. Average grasp-points in Trial 1 and 5 suggests JA typically reorients her grasp towards the weighted end of the object, grasping ≤1.31 cm from the optimum grasp-point. When grasping the right-weighted object, JA deviated to a more extreme rightward grasp; average grasp-point was 4.20 cm further right than the optimum point (6.29 cm) by Trial 5, whereas grasp-points of healthy controls were less than half a centimetre from the optimum point. Observing the grasp-points of JA in relation to the optimum grasp point to compensate for object weight distribution, her point of grasp was further from the optimum point in Trial 5 compared to Trial 1 in the visual-spatial cue condition for both the left and right weighted objects, showing that she continues to adapt her grasp-point even if they were more accurate in previous trials. Similarly, patient JA’s grasps are much more varied suggesting that she does not confidently learn the object-weight associations but may continue to exercise a trial-and-error procedure throughout.

Statistically this behavior was not so much apparent in the average grasp-point variance itself but in the standard deviation of her grasp-point variance. On the average grasp-point variance JA showed marginally significant differences on the memory associated condition (*M* = 20.69 cm) compared to healthy controls (*M* = 12.78 cm, *p* = 0.057, *t* = 1.691; an estimated 94.26% falling below JA’s score) and non-apraxic controls (*M* = 12.92 cm, *p* = 0.055, *t* = 1.798; an estimated 94.50% falling below JA’s score) controls. In the visual-spatial cue condition JA’s grasp-point variance was not different from control participants (healthy controls: *p* = 0.435, *t* = 0.168; non-apraxics: *p* = 0.453, *t* = 0.122). But critically JA did differ in both conditions on the standard deviation of her grasp-point variance. On the memory associated condition JA’s variance standard deviation at 20.20 cm was significantly larger than healthy controls (*M* = 4.52 cm, *p* = 0.018, *t* = 2.333; an estimated 98.18% falling below JA’s score), and non-apraxics (*M* = 4.10 cm, *p* = 0.001, *t* = 4.504; an estimated 99.9% falling below JA’s score). This is similarly evidenced by the standard deviation of patient JA’s grasp-point variance in the visual-spatial cue condition. JA’s grasp-point variance standard deviation at 19.74 cm was significantly greater than healthy controls (*M* = 6.28 cm, *p* = 0.02, *t* = 2.279; an estimated 97.99% falling below JA’s score), and non-apraxic participants (*M* = 5.23 cm, *p* = 0.014, *t* = 2.667; an estimated 98.58% falling below JA’s score). Of course on the neutral-control condition neither JA’s grasp-point variance (*M* = 2.92 cm) nor the standard deviation of patient JA’s grasp-point variance (*M* = 5.80 cm) was different from healthy controls (both not significantly different to JA at *M* = 9.22 cm and *M* = 6.27 cm subsequently) or non-apraxics (both not significantly different to JA at *M* = 11.29 cm and *M* = 3.37 cm subsequently).

### Accuracy Change Across Blocks (BC)

#### Healthy Controls vs. Non-Apraxics

Non-significant main effects and interactions from the two-way mixed model ANOVA confirmed that performance change across Blocks was comparable between control groups: OBJECT, *F*_(1.288,27.045)_ = 0.986, *p* = 0.381, ${\text{η}}_{p}^{2}$ = 0.045, GROUP *F*_(1,21)_ = 0.385, *p* = 0.542, ${\text{η}}_{p}^{2}$ = 0.018, OBJECT × GROUP *F*_(1.288,27.045)_ = 0.264, *p* = 0.671, ${\text{η}}_{p}^{2}$ = 0.012. Both healthy and non-apraxic controls adjusted their point of grasp across blocks depending on the weight distribution of each object; individual scores for performance change over blocks confirms that all healthy and non-apraxic control participants successfully adapted their grasp-point to accommodate for the weight distribution when the objects were reintroduced in later blocks (see Table 5 for performance change over trials, Table 6 for average grasp-points and Figure 3 for accuracy change across blocks); grasps were ≤1.32 cm from the optimum grasp-point by the final block. Accuracy was also maintained across blocks (BC ranged from 0.094 to 0.583).

#### Patient AH

Accuracy change was worse than both healthy and non-apraxic controls during the memory-associated and visual-spatial cue conditions (for all comparisons *p* < 0.05, with at least an estimated 99.65% of controls falling below AH’s score). Patient AH’s score for accuracy change across blocks (BC ≥ 3.24) was indicative of consistently inaccurate object grasps compared to both control groups (BC ≤ 0.583). Average grasp-points confirm that AH did not adjust her grasp according to the weight distribution of each object but maintained a more central grasp; across both Block 1 and Block 5, AH’s grasp-point ranged between 11.50 and 13.45 cm, at least 5.20 cm from the optimum point of grasp. This suggested that AH failed to utilize stored knowledge of weight distribution when the object was reintroduced.

As before, patient AH’s performance change was comparable to healthy (*p* = 0.344, *t* = −0.411; an estimate of 34.38% of controls falling below AH’s score) and non-apraxic controls (*p* = 0.339, *t* = −0.430; an estimate of 33.94% of controls falling below AH’s score) when grasping the neutral-control object. Patient AH’s accuracy was consistently high (BC = 0) and maintained a central grasp-point within 1.48 cm from the optimum point of grasp.

#### Patient GW

Similarly, during the memory-associated and visual-spatial cue conditions patient GW performed worse than healthy controls and non-apraxics; for all comparisons *p* < 0.05, with at least an estimated 96.76% of controls falling below GW’s score. Patient GW grasped each object centrally at least 5.18 cm from the optimum grasp-point resulting in a consistently poor accuracy change across blocks (BC ≥ 4.20).

Mirroring patient AH, when grasping the neutral-control object, GW’s performance change was equivalent to healthy (*p* = 0.344, *t* = −0.411) and non-apraxic controls (*p* = 0.339, *t* = −0.430). Patient GW maintained a central point of grasp within 1.77 cm from the optimum grasp-point confirming that grasps were consistently accurate across blocks (BC = 0).

#### Patient JA

Across all three conditions (memory-associated/visual-spatial cue/neutral-control) patient JA’s performance change was comparable to controls (*p* > 0.05; an estimated 12.60% to 67.27% of controls falling below JA’s score)**.** However, as discussed when examining grasp-point behavior across trials, patient JA makes slow and deliberate movements as if she struggles with the task, evident in a sub-analysis showing abnormal grasp-point variance across trials. The same sub-analysis is also applied here to show that JA exercises a trial-and-error procedure until the final experimental block. When grasping the left weighted object in the memory-associated condition and the right weighted object in the visual-spatial cue condition, grasp-points moved further away from the optimum point of grasp to compensate for weight distribution in Block 5 compared to Block 1 (Table 6). Additionally, the average point of grasp of the left weighted visual-spatial cue condition in Block 1 was on the opposite side of the object from the optimum grasp-point indicating that she did not utilize the dot cue to indicate weight distribution. Therefore, although performance change appears comparable to control groups, patient JA’s grasp behavior demonstrates performance deficits that differentiate her from control groups and may be indicative of more subtle deficits in the integration of visible and known object properties.

## Discussion

To assess whether apraxic patients successfully integrate stored knowledge of objects into action plans, participants were required to learn different weight distributions when lifting and balancing objects using a pincer grip. Over two conditions, each objects’ weight distribution was indicated by either a memory-associated cue (object color) or visual-spatial cue (visible dot over the weighted end). If apraxic patients fail to incorporate stored information into their grasp, we expected that patients might disregard the location of the objects’ center of mass and instead over-rely on visual information, resulting in more centrally oriented grasps based on object structure. The experiment was designed to examine whether patients could learn to grasp the weighted objects accurately when given a meaningful visual-spatial cue indicating the object weight distribution, which would result in increasingly accurate grasps over time if this higher-level information was successfully integrated.

Performance change across trials (TC) and across blocks (BC) in the neutral-control screening condition confirmed that all apraxic patients (AH, GW, and JA) successfully grasped and balanced the evenly weighted object, eliminating the possibility any confounds such as hemispatial neglect or impaired task comprehension might be impacting their performance in the experimental conditions. Comparable to healthy and non-apraxic controls, during consecutive grasps of the neutral-control object (TC) and when grasping the object as it was reintroduced in later blocks (BC), apraxic patients’ central grasp-points remained close to the optimum point of grasp to compensate for weight distribution. Accurate grasping performance during the neutral-control condition indicates that apraxic patients can successfully manipulate objects when the weight distribution is indicated by the objects’ structure (symmetrical cylinder).

Although patient JA’s performance change was within the normal range (see below for a discussion of JA’s pattern of results) during a majority of the memory-associated and visual-spatial cue conditions, patients AH and GW failed to update their grasp-point when the objects were unevenly weighted in both conditions. For both the memory-associated and visual-spatial cue conditions, patient AH and GW maintained a central grasp-point during recurrent trials with the same object (TC) or when the objects were reintroduced in later blocks (BC). Failure to compensate for load torque by reorienting grasps towards the center of mass suggests that these apraxic patients failed to integrate acquired knowledge regarding objects into action plans. Inaccurate grasp-points persisting into the final test block was particularly representative of this. Paired with unimpaired behavior in the neutral-control condition, grasp performance of patients AH and GW suggests an over-reliance on the structural properties afforded by the object. Maintained central grasp-points in the memory-associated and visual-spatial cue conditions perhaps indicate that AH and GW continually referred to structural properties afforded by the object to guide their grasp behavior and did not benefit from either a meaningful visual-spatial cue or symbolic cue of weight distribution.

Patient AH and GW’s performance is compatible with previous research indicating that in addition to impaired perception of skilled object-use (Buxbaum and Saffran, 2002; Buxbaum et al., 2003; Myung et al., 2010), apraxic patients frequently choose inappropriate non-functional grasps (Randerath et al., 2009, 2010; Sunderland et al., 2011) or demonstrate impaired grip force for familiar objects (Gordon et al., 1993; Dawson et al., 2010; Hermsdörfer et al., 2011; Eidenmüller et al., 2014). The performance of patient AH and GW across all three conditions support the proposal that the ventro-dorsal stream is compromised in these patients, resulting in impaired performance when grasping asymmetrically weighted objects. Confirmation that the impairment lies at the ventro-dorsal level comes from the fact that processing of object structure remains intact. Therefore ventro-dorsal disruption appears to impair skilled use of familiar objects, but also when learning to manipulate novel objects.

Interestingly, both patients AH and GW did not appear to benefit at all from the “dot” cue in the visual-spatial cue condition, and there was no evidence of learning. In healthy populations when an object is asymmetrically weighted, grasp-points typically migrate towards the weighted end, particularly when visual cues indicate where the center of mass is located (Endo et al., 2011). Apraxics use of familiar objects also improves from pantomime to actual-use with increased affordance or contextual cues (De Renzi and Lucchelli, 1988; Buxbaum and Saffran, 2002; Sunderland and Shinner, 2007; Goldenberg, 2009; Randerath et al., 2011). Although apraxic patients would not use the visual-spatial cue as effectively as control participants, it was hypothesized that the presence of increased visual information in the form of a visible dot over the weighted end might prompt more appropriate grasps in later trials or when the object was reintroduced.

It is possible that a visual cue, such as a dot, is not ecologically meaningful and subsequently requires more explicit learning. This differs from implicit visual geometric cues of shape and size that are ecologically meaningful (Gentile, 2000; Salimi et al., 2003). Consequently the explicit learning of a visual dot-weight association may also be reliant on higher order perceptual processes to conceptualize the meaning of the dot cue. If this is the case, comparable performance in the memory-associated and visual-spatial cue conditions may be due to both requiring integration of stored and visible information via the ventro-dorsal stream. Therefore, it is reasonable that apraxic patients AH and GW did not benefit from the high-level visual cue. Studies showing improved apraxic performance with increased contextual information may be attributed to an increased presence of low-level affordance cues regarding the objects’ size and structure. Yet, it remains that apraxic patients may be able to register and utilize these memory-associated and visual-spatial cues but that low-level affordance cues are more dominant. According to the affordance competition hypothesis (Cisek, 2007), potential motor actions are generated simultaneously and selected on the basis of the action goal. Therefore, if object affordances compete for selection, the more symbolic memory-associated or visual-spatial cues may be overpowered by more salient low-level cues of object structure. Although it is not certain why these apraxic patients did not benefit from the visual-spatial cue, this observation is interesting when trying to understand what information, be it visual or symbolic, individuals use when manipulating objects to achieve action goals. If apraxic patients are more reliant on low-level affordance cues, this could have a substantial impact on their ability to learn to use new objects or appropriately use familiar objects when these cues are ambiguous. However, as very few studies have assessed learning of skilled movement in apraxia this can only be speculated, and emphasizes the need to explore learning in apraxia to determine the types of cues these patients can successfully utilize to inform their grasp.

Additionally, it was somewhat surprising that patients’ AH and GW did not benefit from short-term sensorimotor feedback to improve grasp performance during subsequent trials within a block (TC). Attributed to the bilateral dorsal stream, rapidly decaying sensorimotor memory is formed and updated with repeated grasps of the same object (Bursztyn and Flanagan, 2008; Buxbaum and Kalénine, 2010). Apraxic patients apply appropriate fingertip force when repeatedly lifting novel objects, suggesting sensorimotor memories can be formed and applied (Gordon et al., 1993; Ietswaart et al., 2001; Dawson et al., 2010; Hermsdörfer et al., 2011; Li et al., 2011; Randerath et al., 2011; Eidenmüller et al., 2014). However, more central grasp-points remained fairly constant between the first and last trial in the current study. AH and GW may fail to update their-grasp points with repeated lifts due to visible structural information and short-term sensorimotor feedback being in conflict; object shape suggests a central weight distribution whereas sensorimotor feedback indicates it is either to the left or the right of the object. In grip force studies, the novel objects were typically symmetrical with a central weight distribution; the shape of the novel object corroborates sensorimotor feedback of object weight, resulting in improved fingertip force with repeated lifts (for examples see Gordon et al., 1993; Dawson et al., 2010; Li et al., 2011). Consequently it is argued that failure to use short-term sensorimotor feedback by patient AH and GW is not because this process is disrupted, but that the design of the current task causes an impediment between visual and sensorimotor information leading to low-level visual affordance cues to be favored. Taken together, the performance of patient AH and GW in memory-associated and visual-spatial cue conditions confirms that they fail to incorporate stored knowledge into action plans even in the presence of certain visible cues.

Interestingly, patient JA’s performance change was comparable to control groups in all conditions, except when compared to healthy controls during repeated grasps (TC) of the visual-spatial cue objects. However, further analyses of grasp-point indicate that patient JA did indeed struggle to apply knowledge-based information or visual-spatial cues in learning to grasp the weighted objects. Exploring JA’s behavior when grasping visual-spatial cued objects, a positive score for accuracy change over trials indicates that JA continued to make errors to the final trial. Although these errors were only minor in contrast to patient AH and GW who consistently failed to adjust their grasp-point according to weight distribution, when examining individual participants’ performance change none of the non-apraxic patients or healthy controls failed to adapt their grasp-point over repeated lifts (TC) and when the objects were reintroduced (BC). Therefore it is possible that apraxic patient JA used compensatory mechanisms to improve performance. Patient JA’s variable grasp behavior also suggests that she may be maintaining a trial-and-error procedure throughout the experiment. In particular, when grasping specific objects within the memory-associated and visual-spatial cue conditions, patient JA’s grasp-point deviated further from the optimum point of grasp to compensate for object weight distribution in later trials and when the objects were reintroduced, whereas control participants grasps moved closer to the optimum grasp-point. Likewise, patient JA’s point of grasp was grossly variable from Block 1–5; JA adjusted her grasp-point by almost 20 cm in both the memory-associated and visual-spatial cue conditions. This behavior seemed to demonstrate a more subtle manifestation of the deficit in the integration of visible and known object properties that results in more changeable grasp accuracy.

These subtle effects in JA were in line with the behavior she displayed. JA, a young and highly motivated patient, performed the task slowly and deliberately. She appeared more aware of her deficit than the other patients. Perhaps this due to the fact that she was aware of her apraxic symptoms that included actual object-use (evident in standard apraxia screening). If this is the case, JA is more likely to compensate for her impairment resulting in improved grasping performance compared to the other apraxic patients. Although patient AH has a similar lesion to JA, she inevitably will have been less aware of her apraxic symptoms that did not include actual object-use. Likewise, GW demonstrated more severe apraxic errors across the screening tasks and may be less able to effectively compensate for his impairment. No compensative strategies in performance of the experimental task were apparent in AH or GW who performed the task very quickly, immediately reaching for the object at the start of each trial and rapidly lifting each object before returning it to the table. In contrast, JA showed awareness of difficulty with the task, commenting on completion that she tried to apply strategies: she said that when the object was placed in the testing area, she observed whether one end of the object landed on the table first as a potential clue to its weight distribution. Although the availability of such cues were avoided through careful placement of each object, it may be beneficial to occlude participants’ view when objects are placed on the table. However, it was felt that the presence of each object during testing ensured that participants were aware that each object reintroduced in later blocks was the same as those seen previously. Finally, the less gross errors of patient JA on the grasping task compared to AH and GW cannot be attributed to better comprehension, as JA scored the least in the language comprehension test. Likewise, JA did not suffer from milder apraxic symptoms; as described, patient GW demonstrated the more severe apraxic symptoms whereas JA’s apraxic behavior was comparable to AH.

Rather than ventro-dorsal processing remaining intact in patient JA, it is believed that through her careful performance, she managed to assemble compensatory strategies, even when weight distribution was afforded by a high-level visual-spatial cue. Appropriate performance when behavior is delayed in apraxic patients suggests that stored knowledge is maintained, but difficult to access. As described, accurate memory-driven reach and grasp performance is observed when apraxic patients pick up basic blocks based on simple size and distance information (Ietswaart et al., 2001). Myung et al. (2010) also confirmed that during semantic judgements, apraxic patients also showed greater fixations on object pictures that were manipulation-related to the target word (e.g., “typewriter” and “piano”) when the manipulation relationship was not task relevant; the fixation position was comparable to their non-apraxic control group but the effect emerged later, again indicating that stored representations are preserved but not easily accessible. The magnitude of delayed activation of manipulation related action information in apraxia is predicted by poorer object-use pantomime performance and the extent to which inferior parietal and posterior temporal regions were compromised (Lee et al., 2014). Therefore, the extended delay between reach and grasp movements used by JA in her slow and deliberate performance (compared to patient AH and GW who initiated grasps immediately) may have enabled her to incorporate stored knowledge into action plans. Further, the variable nature of her points of grasp along each object may be indicative of when her compensatory strategies were less effective. This may also indicate why JA continued to make grasping errors by the final trial when grasping the visual-spatial cued objects.

Although the design of the current study delayed reach-to-grasp action between trials by requiring participants to return their hand to the table before beginning another grasp movement, the duration of this delay was not controlled. Further investigation is required to confirm whether delay between reaching and grasping can reduce performance errors when balancing novel objects. It is probable that such compensatory strategies may rely on critical brain structures being intact; JA presented with frontal lesions that implicate white matter whilst parietal regions remain undamaged (as was the case in AH). In contrast, GW’s lesion implicates temporal and parietal regions of the left hemisphere suggesting that the critical juncture between the ventral and dorsal pathways may be compromised (Rizzolatti and Matelli, 2003; Buxbaum and Kalénine, 2010; Rizzolatti et al., 2011; Binkofski and Buxbaum, 2013; Vingerhoets, 2014). This corresponds with patient GW’s markedly poor performance across all apraxic tests. Based on research showing a strong association between impaired object-use and temporal and parietal damage (Goldenberg, 2009; Vingerhoets, 2014), impaired use of memory-associated and visual-spatial cued information is expected in this patient.

In conclusion, apraxia was associated with a disrupted ability to utilize memory-associated or visual-spatial cued information indicating weight distribution. Specifically, patient AH and GW failed to successfully incorporate memory-associated information where weight distribution was indicated by the objects color, and visual-spatial cued information in the form of a dot cue over the objects weighted. Grasps were inaccurate during repeated lifts and when the objects were reintroduced. A third apraxic patient (JA) seemed to compensate for these difficulties but still showed performance errors that may be attributable to a more subtle impairment. These results indicate that apraxia impairs the ability to utilize meaningful visual-spatial cue or symbolic memory-associated cues when grasping objects to achieve specific action goals. Crucially, the abnormal grasping behavior in these apraxic patients suggests that integration of visible and known object properties attributed to the ventro-dorsal stream is impaired. Not only does disruption to ventro-dorsal processing impair use of familiar objects, but also these results would predict that apraxia is associated with difficulty learning to manipulate new objects.

## Author Contributions

CE, conception and design of the research task; acquisition, analysis and interpretation of the data; drafting the manuscript and final editing. MGE, contribution to the conception of the task, critically revising and editing the manuscript, and final approval of the manuscript. LJT, contribution to the conception of the task, final approval of the manuscript version to be published. MI, substantial contribution to the conception and design of the research task, interpretation of the data and critically revising and editing the manuscript.

## Conflict of Interest Statement

The authors declare that the research was conducted in the absence of any commercial or financial relationships that could be construed as a potential conflict of interest.

## Footnotes

^1^http://www.mccauslandcenter.sc.edu/mricro/mricron/

^2^http://www.mricro.com/clinical-toolbox/
